# Anxiety, financial stress, and childhood trauma are associated with psychotic-like experiences during the postpartum period

**DOI:** 10.3389/fpsyt.2025.1586471

**Published:** 2025-09-02

**Authors:** Rebecca E. Grattan, Sophie London, Matthew D. Hammond

**Affiliations:** School of Psychological Sciences, Te Herenga Waka, Victoria University of Wellington, Wellington, New Zealand

**Keywords:** postpartum mental health, psychotic like experiences, subclinical psychosis, mental health, maternal

## Abstract

**Purpose:**

Psychotic-like experiences (subclinical psychosis symptoms) predict the development of severe mental distress in the general population and can also be an experience of clinical concern. Little attention has been paid to psychotic-like experiences occurring for women during the postpartum period, despite it being a time of elevated mental distress. In particular, it is unknown how these symptoms present across the postpartum period and what risk factors are associated with them.

**Methods:**

The present study examined psychotic-like experiences across two time points, 4 weeks apart, during the first 6 months postpartum. The prevalence of psychotic-like experiences and the relative change over time were examined, including common risk factors for psychotic-like experiences outside the postpartum period. The included risk factors were history of childhood trauma, birth trauma, depression, anxiety, financial stress, postpartum stress, and sleep difficulties. Social support was also included as a possible protective factor.

**Results:**

Psychotic-like experiences were generally prevalent and persistent over time in the postpartum period and were associated with distress. Anxiety symptoms, financial stress, and history of childhood trauma were associated with psychotic-like experiences cross-sectionally. There was no evidence that any commonly understood risk factors accounted for the change in psychotic-like experiences over 4 weeks.

**Conclusions:**

These findings indicate that stress and anxiety may contribute to the development of postpartum psychotic-like experiences, but it is unclear what factors may lead to worsening symptoms over time.

## Highlights

- Subclinical psychosis symptoms are prevalent and persistent in the postpartum period.
- Subclinical psychosis symptoms are associated with anxiety, financial stress, and childhood trauma.
- There may be clinical utility to measuring postpartum subclinical psychosis experiences.

## Introduction

The postpartum period is a time of increased mental distress, affecting up to 20% of women (1). Postpartum mood disorders are very common (2), and suicide is a leading cause of maternal mortality (3). Experiences of psychosis are also common in the postpartum period, with incidence ranging from 0.86 to 2.6 per 1,000 births (4). Most work focused on mental health during the postpartum period has taken a diagnostic approach, which limits our understanding in several ways. The nosology of postpartum psychosis is not well understood (5). While some consider it a manifestation of bipolar disorder, there are also arguments that it may be a distinct but related entity (6). The timing of diagnoses of postpartum psychosis is also unclear. In the *Diagnostic and Statistical Manual of Mental Disorders*, postpartum psychosis is not recognized as a distinct condition but can be diagnosed as a brief psychotic disorder if it occurs within 4 weeks postpartum (7), despite episodes of psychosis occurring outside of this time (5). The timing and nosology of postpartum depression are also debated (8). Therefore, taking a more dimensional approach to understand postpartum mental health may be helpful. Subclinical psychotic experiences are a transdiagnostic risk factor for severe mental distress in the general population (9) and also predict suicide ideation and behavior (10). Despite this, there has been little interest in understanding their role in the postpartum period.

Psychosis symptoms occur across a spectrum (11). Experiences at the lower severity end of this spectrum (often called subclinical) can present as distressing and indicate general mental health difficulties or can be non-distressing and considered normative (11). Investigating subclinical presentations of psychosis is vital. Subclinical psychosis symptoms occur transdiagnostically and can indicate that someone is at high risk for developing a range of severe mental disorders, in particular psychotic disorders, but also other difficulties such as depression and bipolar disorder (12). Such presentations are also helpful to understand, as they may be lower-level manifestations of the same mechanisms seen in clinical disorders, such as those seen in clinical staging models (13). Therefore, the study of these presentations can aid preventative work (14). Such symptoms can also occur outside of clinical diagnoses but may still be a major source of distress and require clinical input (15). Given that the postpartum period is a time of elevated risk for mood and psychosis presentations, it would be helpful to understand how subclinical psychotic symptoms may present dimensionally during this time period and what risk factors are associated with increased psychotic symptoms.

Psychotic-like experiences (PLEs) are a form of subclinical psychosis that occurs in the general population starting childhood (10, 16). PLEs are perceptual aberrances and delusion-like thoughts, similar to full-threshold positive psychotic symptoms, but are less severe, for example, thinking you heard your name called when there was no one there. PLEs are used in the measurement of clinical high-risk syndromes and are also linked to other poor mental health factors such as suicide ideation and behavior (10), self-harm (17), sleep difficulties (18), and poorer functioning (19).

Two studies have examined PLEs in the postpartum period. One assessed PLEs during pregnancy and postpartum in 101 women, indicating that 59% of the participants endorsed one or more PLEs during the postpartum period and 10% endorsed at least one PLE as highly distressing (20). Postnatal PLEs were associated with depression symptoms, fear during delivery, and being unemployed, but not other risk factors such as sleep and anxiety about childbirth (20). This study did not measure PLEs longitudinally after birth. Another cross-sectional study in a larger population of 1,085 participants found that 93.58% of participants experienced at least one PLE, and while delusion-type PLEs were associated with traumatic birth experiences, hallucination-type PLEs were not (21). In sum, the current picture indicates that PLEs are prominent during the postpartum period, with a decent percentage associated with severe distress. Further longitudinal research is needed to better understand the longitudinal presentations of PLEs across the postpartum period and to identify which risk factors are associated with such PLEs.

The present study examined PLEs across two time points during the postpartum period to examine how postpartum PLEs occur over time, which risk factors may predict the initial PLEs, and which risk factors may predict relative change in PLEs. The included risk factors were history of childhood trauma, birth trauma, depression, anxiety, financial stress, postpartum stress, and sleep difficulties. Social support was also included as a possible protective factor.

## Method

### Participants and procedure

A total of 475 participants were recruited through targeted social media advertisements (Facebook and Instagram posts to parents of children under 12 months living in New Zealand) and advertising through community parental groups (e.g., Plunket) between November 2022 to November 2023. Eligible participants were people aged 18+ living in New Zealand who had given birth 6 weeks to 6 months prior and who consented to participate. The participants completed two online surveys at Time 1 and then 4 weeks later (Time 2). This design allowed us to understand how PLEs occur over time and whether risk factors uniquely predict later PLEs. Participants were excluded from analyses due to dropout (*N* = 189), being more than 6 months postpartum at Time 1 (*N* = 26), or not completing more than two surveys at Time 1 (*N* = 37).

The final sample of 223 people included 217 people who identified as cisgender female and one who identified as non-binary (one participant selected *preferred not to say*, and four participants did not answer). The participants ranged from 0 to 6 months postpartum (M = 2.74, SD = 1.73) and were aged from 20 to 44 (M = 30.23, SD = 4.68). The most common ethnic identities included New Zealand European (n = 174), Māori (n = 34), Asian (n = 21), Pasifika (n = 5), and other European (n = 26). Excluded participants’ psychotic-like experiences total scores were not significantly different from those of the included sample. However, the excluded participants (M = 8.02, SD = 10.24) did significantly differ on psychotic-like experiences distress scores, reporting higher scores than the included sample (M = 6.21, SD = 7.13) at Time 1 (*t*(388.70) = −2.15, *p* = .03). The magnitude of this difference in means (mean difference = −1.81, 95% CI = −3.46 to −0.16) was small (Cohen’s *d* = 0.21). This study was approved by the Victoria University of Wellington Human Ethics Committee (#30643). Our predictions and analytic plan were preregistered (osf.io/v3bgq).

### Measures

The participants completed the following measures at both time points.

#### Psychotic-like experiences

The Prodromal Questionnaire-16 (22) includes two scores. We excluded two items from the scale due to similarity with predictor variables (i.e., “I feel uninterested in the things I used to enjoy” and “I get extremely anxious when meeting people for the first time”). First, *total psychotic-like experiences* was the sum of a possible 14 experienced events [e.g., “I often hear unusual sounds like banging, clicking, hissing, clapping or ringing in my ears”; (Yes/No)]?. Second, for each experienced event, participants also rated their *psychotic-related distress*, which was the sum of the extent to which they felt “frightened, concerned or it causes problems for me” (1 = *strongly disagree* to 5 = *strongly agree*). Psychotic-like experiences (α = .70) and psychotic-related distress (α = .70) indicated acceptable reliability.

#### Sleep quality

The 14-item *Postpartum Sleep Questionnaire* (PSQ; 23) was used. Items were rated for how often they occurred in the past week (e.g., “In the past week, how often did I experience reduced sleep due to infant care in the middle of the night”; 0 = *never*, 1 = *few*, 2 = *sometimes*, 3 = *often*, and 4 = *almost always*) and were summed (α = .88).

#### Stress

The 30-item *Maternal Postpartum Stress Scale* (24) was used. Items asked about stress due to different factors in the past month, and the items (e.g., “Baby’s development”; 0 = *very low or no stress at all* to 4 = *very high stress*) were summed (α = .88).

#### Financial stress

The seven-item *Financial Stress Scale* asked participants to rate if they had experienced any of the seven items in the last year (25; e.g., “Could not fill or collect prescription medicine”). Items were summed (α = .80).

#### History of childhood trauma

The *Adverse Childhood Experiences Questionnaire* (ACES-Q; 26) assessed participants’ past experiences of trauma prior to age 18. Responses to 17 possible events (e.g., “Did a parent or other adult in the household often or very often act in a way that made you afraid that you would be physically hurt?”) were summed (α = 87).

#### Social support

The *Postpartum Social Support Questionnaire* (PSSQ; 27) assessed perceptions of social support. Responses to the three items were summed (e.g., “In general, do you feel your (partner, parent figure, other family/friends) have been supportive”; 0 = *never* to 4 = *always*). Items indicated relatively low but acceptable reliability given the few items (α = .62).

#### Depression

An adapted version of the Edinburgh Postnatal Depression Scale (28) was used. Two items were removed due to measurement overlap (29) with the anxiety measure (e.g., “I have felt scared or panicky for no very good reason”). The remaining eight items indexed the presence and severity of depressive symptoms in the past week (e.g., 0 = *as much as I always could* to 3 = *not at all*). Items were summed and indicated good reliability (α = .88).

#### Anxiety

The 21-item *Beck Anxiety Inventory* (30) measured physical anxiety symptoms. Items indexed the presence and severity of anxiety symptoms in the past month (e.g., “Numbness or tingling”; 0 = *not present*, 1 = *mildly, but it didn’t bother you much*, to 3 = *severely, it bothered you a lot*). Items were summed and indicated excellent reliability (α = .91).

#### Birth trauma symptoms

The 22-item City Birth Trauma Scale (CBTS; 31) was used. Items indexed the frequency of symptoms of trauma related to the birth in the last week (e.g., “Recurrent unwanted memories of the birth”; 0 = *not present*, 1 = *once*, to 3 = *5 or more times*). Items were summed and indicated excellent reliability (α = .93).

### Data analysis

We first examined the prevalence of PLEs at each time point and relationships between PLEs and risk factors. We conducted summary statistics, including means for each measure and correlations. We then conducted within-person t-tests to estimate the average level of change across time. Finally, to understand 1) whether PLEs or PLE distress were associated with common concurrent risk factors and 2) whether common risk factors predicted a change in PLEs or PLE distress, hierarchical regression analyses were conducted in Mplus (version 8.7; 32) using Full Information Maximum Likelihood and numerical integration estimation (33). Predictors were grand-mean centered.

## Results

### Summary statistics

Descriptive statistics and Pearson’s correlations are presented in **Table 1**. At Time 1, 71.3% of participants (n = 159) reported at least one PLE; at Time 2, 63.6% reported at least one PLE (n = 142). Experiencing more psychotic-like experiences was highly correlated with psychotic-related distress. High correlations between Time 1 and Time 2 indicated that the relative levels of psychotic-like experiences and psychotic-related distress were generally stable across the 4-week timespan. Within-person t-test indicated that people exhibited significant and small decrease in psychotic-like experiences over the 4 weeks [*average difference* = .27, 95% confidence interval for the difference (95% CI) = 0.01 to 0.53, *t* = 2.02, *p* <.05, Cohen’s *d* = 0.14], while there was no evidence for change in psychotic-related distress (*average difference* = .82, 95% CI = −0.06 to 1.69, *t* = 1.85, *p* = .07, *d* = 0.13).

**Table 1:** Descriptive statistics and correlations for study measures.

Measure	Mean (SD)	*1.*	*2.*	*3.*	*4.*	*5.*	*6.*	*7.*	*8.*	*9.*	*10.*	*11.*	*12.*
1. Psychotic-like experiences (Time 1)	2.0 (2.1)	–											
2. Psychotic-like experiences (Time 2)	1.8 (2.2)	.59^**^	–										
3. Psychotic-related distress (Time 1)	6.2 (7.1)	.95^**^	.60^**^	–									
4. Psychotic-related distress (Time 2)	5.5 (7.5)	.56^**^	.95^**^	.60^**^	–								
5. Sleep quality	30.1 (10.0)	.28^**^	.25^**^	.30^**^	.26^**^	–							
6. Stress	44.2 (17.5)	.25^**^	.21^**^	.32^**^	.27^**^	.64^**^	–						
7. Financial stress	1.1 (1.7)	.36^**^	.24^**^	.38^**^	.24^**^	.26^**^	.33^**^	–					
8. History of childhood trauma	3.3 (3.7)	.37^**^	.24^**^	.37^**^	.23^**^	.29^**^	.27^**^	.51^**^	–				
9. Social support	11.3 (2.7)	−.21^**^	−.15^*^	−.22^**^	−.20^**^	−.33^**^	−.43^**^	−.25^**^	−.34^**^	–			
10. Depression	8.1 (4.7)	.29^**^	.22^**^	.39^**^	.30^**^	.57^**^	.73^**^	.34^**^	.30^**^	−.32^**^	–		
11. Anxiety	13.8 (10.4)	.42^**^	.30^**^	.49^**^	.33^**^	.49^**^	.55^**^	.34^**^	.38^**^	−.32^**^	.56^**^	–	
12. Birth trauma symptoms	17.7 (14.2)	.36^**^	.27^**^	.44^**^	.33^**^	.53^**^	.65^**^	.44^**^	.42^**^	−.32^**^	.72^**^	.66^**^	–

*p <.05; **p <.01.

### Cross-sectional hierarchical model

The first analysis examined the cross-sectional data gathered at Time 1. Psychotic-like experiences were regressed on sleep quality, stress, financial stress, history of childhood trauma, social support, depression, anxiety, and birth trauma symptoms at Time 1. Length of time postpartum was included as a covariate. In this model (table included in the **Supplementary Material**), greater anxiety was a moderately strong predictor of greater psychotic-like experiences (*unstandardized B* = .05, 95% CI = 0.02 to 0.09, *t* = 3.16, *p* <.01, *β* = .26). Greater financial stress (*B* = .20, 95% CI = 0.03 to 0.36, *t* = 2.30, *p* = .02, *β* = .16) and history of childhood trauma (*B* = .08, 95% CI = 0.00 to 0.15, *t* = 1.99, *p* = .047, *β* = .13) were small predictors of greater psychotic-like experiences. No other predictors were significant (*β*s = −.07 to.08, *p*s >.40). Next, the same regression model was conducted with psychotic-related distress as the outcome. Greater anxiety was a moderately strong predictor of greater psychotic-related distress (*B* = .21, 95% CI = 0.10 to 0.33, *t* = 3.74, *p* <.01, *β* = .31), and financial stress was a small predictor of greater distress (*B* = .61, 95% CI = −0.05 to 1.17, *t* = 2.12, *p* = .03, *β* = .15). No other predictors were significant (*β*s = −.05 to.11, *p*s >.08). In sum, after adjusting for the multiple overlaps between risk factors and protective factors, people’s anxiety and financial stress both emerged as predictors of greater psychotic-like experiences and greater psychotic-related distress.

### Longitudinal hierarchical model

The second set of models was our pre-registered hypothesis tests. We examined the extent to which common risk factors predicted residualized changes in 1) PLE or 2) PLE distress between Time 1 and Time 2. We regressed psychotic-like experiences at Time 2 on psychotic-like experiences at Time 1 (which accounted for the generally stable features over time), as well as sleep quality, stress, financial stress, history of childhood trauma, social support, depression, anxiety, birth trauma symptoms, and length of time postpartum. The results from the model examining predictors of change in PLE and PLE distress over time are displayed on the left side of **Table 2**. The very strong and significant associations between the respective predictors at Time 1 and the corresponding outcome at Time 2 indicated high consistency in these symptoms across time, even after adjusting for the other measures (see first row of **Table 2**). However, there was no evidence that any of the predictors accounted for the future variance in psychotic-like experiences. The results for PLE distress were of an identical pattern. Psychotic-related distress was relatively consistent across time, and there was no evidence that any of the hypothesized predictors accounted for the remaining variance in their experienced distress. To summarize, there was a general pattern of decrease in psychotic-like experiences over the study period and no evidence for average change in distress over the postpartum period. No evidence emerged to suggest that any of the theorized correlates (e.g., stress, anxiety, and traumatic experiences) accounted for variance in this change over time. In other words, people who were higher in PLE and PLE distress at Time 1 were also higher at Time 2.

**Table 2:** Results from regression models predicting the residualized change in postpartum birthing parents' (*N* = 223) psychotic-like experiences (center side) or psychotic-related distress (right side) over 4 weeks.

Predictors	Psychotic-like experiences (Time 2)				Psychotic-related distress (Time 2)			
	*B*	95% CI *Lo.*, *Hi.*	*β*	*p*	*B*	95% CI *Lo.*, *Hi.*	*β*	*p*
Experience/distress measure at Time 1	.56	0.40, 0.72	.55	<.001	.61	0.41, 0.78	.57	<.001
Sleep quality (Time 1)	.02	−0.03, 0.06	.07	.55	.04	−0.14, 0.22	.05	.67
Stress (Time 1)	.00	−0.03, 0.04	.01	.96	.01	−0.13, 0.15	.03	.88
Financial stress (Time 1)	.03	−0.18, 0.24	.02	.79	.02	−0.82, 0.85	.00	.97
History of childhood trauma (Time 1)	−.01	−0.11, 0.10	−.01	.89	−.05	−0.45, 0.35	−.03	.80
Social support (Time 1)	.00	−0.14, 0.14	.00	.98	−.10	−0.61, 0.41	−.04	.69
Depression (Time 1)	−.01	−0.11, 0.10	−.02	.89	.01	−0.35, 0.37	.01	.95
Anxiety (Time 1)	.00	−0.05, 0.06	.02	.90	−.01	−0.19, 0.17	−.02	.91
Birth trauma symptoms (Time 1)	.01	−0.03, 0.04	.03	.80	.02	−0.11, 0.15	.04	.77
Age (Time 1)	.00	−0.07, 0.08	.01	.91	.02	−0.28, 0.33	.02	.88

*B* represents unstandardized parameter estimate*. 95% CI Lo.* and *Hi.* represent estimated values for the lower and higher bounds of 95% confidence intervals for model parameters, respectively. *β* represents standardized parameter estimate indicating the size of the effect.

## Discussion

The findings indicated that postpartum PLEs and PLE distress were common and generally stable across the 4-week postpartum period. The occurrence of postpartum PLEs in the present sample (71.3%) was more common than another postpartum study (52%–59%) using questionnaire assessment of subclinical psychosis (20) but less common than the prevalence of symptoms (93.58%) identified by a study using diary-based methods for subclinical psychosis (21). To our knowledge, our study is the first to investigate change in PLEs across 4 weeks during the first 6 months postpartum.

Postpartum PLEs exhibited small decreases over time on average but were nonetheless relatively stable over 4 weeks. In addition, PLE-related distress was also persistent over time. Generally, more persistent subclinical experiences indicate a higher severity than transient subclinical psychotic experiences, as shown by higher stress reactivity (34) and higher risk for developing clinical mental disorders such as a psychotic disorder (14). This indicates that further investigation of postpartum PLEs is warranted for the prevention of mental health difficulties in the postpartum period. Importantly, our analyses indicate the general patterns of PLEs in our participant group and do not preclude the possibility that specific people experienced particularly increasing (or decreasing) patterns of PLEs, as has been seen in other populations, such as in children and adolescents (35).

In contrast to past studies, we did not find that depression (20) and childbirth trauma (21) were significant predictors of PLEs or PLE distress, once accounting for the variance explained by other common risk factors. Instead, concurrent anxiety symptoms, financial stress, and childhood trauma emerged as the most important factors cross-sectionally. The association found between financial stress and PLEs postpartum supports recent findings that social determinants are important for understanding psychosis risk (36) and perinatal mental health (37). Historically, research has shown that people experiencing poverty are at higher risk for subclinical psychotic symptoms (38). However, these patterns may be better explained by other factors, such as higher neighborhood crime (39). Our study did not find a relationship with PLEs and current postpartum stress. It may be that relatively historic stress factors, such as finances and childhood trauma, are better predictors of postpartum psychotic experiences than current stress, consistent with evidence from similar populations linking both historical and prenatal stress to postpartum psychotic relapse (40). This suggests that historic brain changes prior to birth may be contributing to the likelihood of experiencing PLEs in the postpartum period. Our work also supports findings that anxiety symptoms are consistently associated with psychosis presentations, including subclinical symptoms in correlational studies (41), and there is some evidence that anxiety may explain links between trauma and some psychotic symptoms in the general population (42). Postpartum anxiety has been addressed less frequently than postpartum depression, despite being common (43).

Unexpectedly, none of the examined risk factors explained changes in PLEs or PLE distress over the 4-week postpartum period. Given the small average decrease and higher relative stability of PLEs over time, perhaps the effects of possible risk factors are only observable across longer periods of time. However, it is also possible that these risk factors are associated with a general predisposition to experience PLEs during the postpartum period but are not mechanisms that contribute to worsening PLEs. It is also possible that increasing PLEs is not a helpful measure for distress in the postpartum period. Risk factors predicting changes in PLEs are poorly understood across populations, but some possible mechanisms linked to changes in psychotic symptoms include negative life events and stress sensitivity in young adults (44) and neural and cognitive impairments during middle childhood (45). As such, suitable indicators for future investigation will likely be individual factors encompassing the ability to cope with stress, and cognitive and neural functioning, alongside environmental indicators of stress.

This is the first longitudinal examination of postpartum PLEs and the first examination of risk factors associated with the occurrence of postpartum PLEs as well as change in PLEs over time. A strength of this study was examining the risk factors concurrently, rather than each independently. These insights can support us in understanding how dimensional postpartum mental health symptoms present. The next steps include investigations of trajectories of PLEs in the postpartum period and longitudinal investigations observing symptoms throughout pregnancy and the early postpartum period. This will assist with understanding how PLEs may predict transdiagnostic clinical mental disorders and how PLEs contribute to impaired functioning during this period, providing further clinical utility.

The possible limitations of the study include that the sample participants responded to advertisements about postpartum mental health and thus may be an oversampling of those experiencing mental health distress. Data were collected from an online survey, allowing access to a broad postpartum population and anonymity for sensitive data collection; however, this did result in some participant dropout at Time 2. The excluded participants experienced slightly higher psychosis-related distress at Time 1 than the included participants. Thus, our eligible sample was less likely to include people with heightened PLEs. Finally, measures were self-reported symptoms, and while these are considered an accurate measure of psychotic experiences (46), additional information may be gained from clinician-administered measures in future research.

These findings have several possible implications. Given that PLEs are common and distressing during the postpartum period, it would be helpful to understand if these contribute to other mental health difficulties such as depression and perinatal suicide, as is seen in the general population (47). Interestingly, worsening PLEs were not predicted by usual risk factors for PLEs, and therefore, this study indicates that we need to better understand what factors may contribute to subclinical psychosis experiences during the postpartum period. There may be perinatal-specific risks involved, such as hormones or specific types of stressors. Finally, given that PLEs are generally linked with higher risk for psychotic disorder (14), as well as other clinical mental disorders, it may be clinically informative to measure these symptoms during pregnancy and postpartum to monitor ongoing postpartum psychosis and mental health risk.

In conclusion, this study suggests that PLEs are generally persistent and prevalent in the postpartum period and are associated with distress, thus indicating that they are an important phenomenon to examine. Anxiety symptoms, financial stress, and history of childhood trauma were associated with postpartum PLEs, but the change in PLEs was not associated with any known risk factors. Future research is needed to better understand the role of PLEs during the postpartum period.

## Data Availability

The raw data supporting the conclusions of this article will be made available by the authors, without undue reservation.
