# Supplementary material for: Anxiety, financial stress, and childhood trauma are associated with psychotic-like experiences during the postpartum period
Source: Front Psychiatry. 2025 Sep 2;16:1586471. doi: 10.3389/fpsyt.2025.1586471 (PMC12441800; doi:10.3389/fpsyt.2025.1586471)
Supplement: Supplementary file 1 [file Table1.docx]

**Supplementary Table 1**

Results from regression models predicting postpartum psychotic-like experiences (left side) or psychotic-related distress (right side) at Time 1 (*N* = 223).

|  | **Psychosis-like Experiences (Time 1)** | | | |  | **Psychosis-related Distress (Time 1)** | | | |
| --- | --- | --- | --- | --- | --- | --- | --- | --- | --- |
| **Predictors** | *B* | 95% CI *Lo., Hi.* | *β* | *p* |  | *B* | 95% CI *Lo., Hi.* | *β* | *p* |
| Sleep Quality (Time 1) | .02 | –.02, .05 | .06 | .40 |  | .02 | –.10, .12 | .02 | .83 |
| Stress (Time 1) | -.01 | –.03, .01 | -.07 | .52 |  | -.03 | –.11, .04 | -.08 | .45 |
| Financial Stress (Time 1) | .20 | .06, .33 | .16 | .02 |  | .61 | .14, 1.08 | .14 | .03 |
| History of Childhood Trauma (Time 1) | .08 | .01, .14 | .13 | .05 |  | .21 | .01, .40 | .11 | .08 |
| Social Support (Time 1) | -.01 | -.09, .08 | -.01 | .86 |  | –.00 | –.29, .29 | –.01 | .99 |
| Depression (Time 1) | .01 | –.07, .09 | .03 | .81 |  | .18 | –.07, .43 | .12 | .23 |
| Anxiety (Time 1) | .05 | .03, .08 | .26 | .00 |  | .21 | .12, .31 | .30 | .00 |
| Birth Trauma Symptoms (Time 1) | .00 | –.02, .02 | .02 | .86 |  | .03 | –.04, .10 | .07 | .44 |
| Age (Time 1) | -.03 | –.08, .03 | -.06 | .46 |  | -.08 | –.27, .11 | -.05 | .50 |

*Note.* *B =* Unstandardized parameter estimate*. 95% CI Lo.* and *Hi.* represent estimated values for the lower and higher bounds of 95% confidence intervals for model parameters respectively. *β* = Standardized parameter estimate indicating the size of the effect.
